# Dr. W. B. Sprague

**Published:** 1891-04

**Authors:** 


### Obituary.

Dr. W. B. Sprague.—In the death on Monday, March 16, 1891, of Dr. William Bishop Sprague, of Pavilion, Genesee county loses one of its best-known and most skilful physicians while yet in the prime of life. The deceased, who was born in 1836, in the town where he died, and was educated in the local schools and at Temple Hill Academy, Geneseo, was a graduate of the Buffalo Medical College in the Class of 1857. He served for a time as resident physician at St. Mary's Hospital, Rochester, removing from there to Oneida county and thence successively to York, Livingston county and Pavilion, succeeding to the practice of his father, Dr. William M. Sprague, on his death. A member of the New York State Medical Association and president of the medical society of his county, he was a man of literary tastes and a clever writer, his contributions appearing in various papers, in *Harper's Magazine*, and in many other periodicals. His disease was kidney trouble. He had never married, and leaves a mother ninety-two years of age, and a sister—Mrs. Celia Lewis, of Pavilion. His funeral was largely attended on Tuesday.

